# The role of soil chemical properties and microbial communities on *Dendrocalamus brandisii* bamboo shoot quality, Yunnan Province, China

**DOI:** 10.3389/fmicb.2025.1551638

**Published:** 2025-04-30

**Authors:** Qian Chen, Jianjie Cao, Manyun Zhang, Lei Guo, Negar Omidvar, Zhihong Xu, Chaomao Hui, Weiyi Liu

**Affiliations:** ^1^Research Institute of Bamboo and Rattan, Cluster Bamboo Engineering Technology Research Center, College of Forestry, Southwest Forestry University, Kunming, China; ^2^College of Resources and Environment, Hunan Agricultural University, Changsha, China; ^3^Centre for Planetary Health and Food Security, School of Environment and Science, Griffith University, Brisbane, QLD, Australia; ^4^College of Land and Environment, Shenyang Agricultural University, Shenyang, China

**Keywords:** soil nutrients, soil microorganisms, nutritional quality of bamboo shoots, microbial communities, *Dendrocalamus brandisii*

## Abstract

**Objective:**

To explore the effects of soil nutrients and microbial communities on the quality of *Dendrocalamus brandisii* shoots in different regions, providing a scientific basis for their development and utilization.

**Methods:**

Using seven different geographic sources of *D. brandisii* from Yunnan Province as research subjects, this study employs chemical analysis and high-throughput sequencing to reveal the relationship between soil nutrients, microbial functional groups, and the nutritional quality of bamboo shoots.

**Results:**

The results indicate that there are significant differences in soil nutrient content among the regions (*p* < 0.05), with bamboo shoots from Baoshan Changning (CN) exhibiting the best overall nutritional quality. The key factors influencing bacterial community changes include pH, available phosphorus (AP), and available potassium (AK). In contrast, the main factors affecting fungal community changes are pH, soil organic matter (SOM), available potassium (AK), and total nitrogen (TN). This version maintains clarity and logical flow, making it easier for readers to understand the different factors influencing bacterial and fungal community changes. The diversity indices of soil microbial communities among different sources of *Dendrocalamus brandisii* show significant differences (*p* < 0.05). The dominant groups in the seven regions include Proteobacteria, Acidobacteriota, Actinobacteriota, Chloroflexi, Ascomycota, and Basidiomycota. The soil microbial community in Baoshan Changning (CN) shows significant structural differences compared to the other six regions, with the highest relative abundances of Chloroflexi and Acidobacteriota. In contrast, the highest relative abundance of Proteobacteria is found in Honghe Shiping (SP), while Actinobacteriota has the highest relative abundance in Yuxi Xinping (XP). RDA analysis indicates that soil nutrients (SOM, pH, AP, TN) affect the water content, soluble sugar, and crude fat of bamboo shoots. Additionally, the bacterial communities including Actinobacteriota, Chloroflexi, Patescibacteria, GAL15, and Cyanobacteria influence the water content, soluble sugar, ash content, protein, and lignin of bamboo shoots.

**Discussion:**

In the fungal community, Basidiomycota, Kickxellomycota, Mucoromycota, unclassified-k-Fungi, and Glomeromycota affect the water content and tannin levels in bamboo shoots. In summary, soil nutrients and soil microorganisms are interconnected and work together to influence the quality of bamboo shoots.

## Graphical abstract

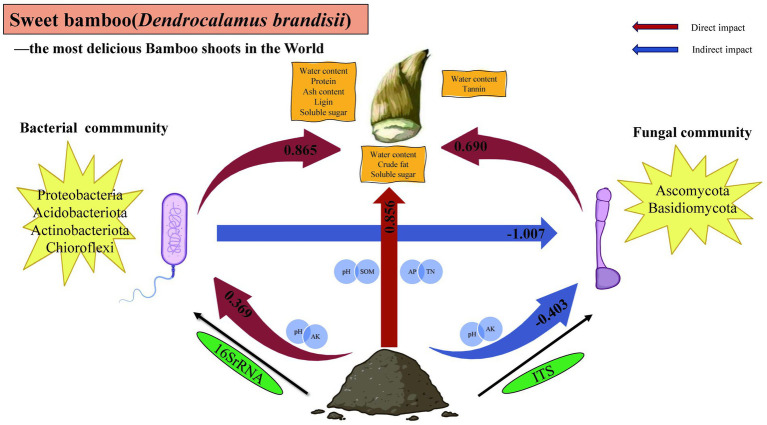

## 1 Introduction

*Dendrocalamus brandisii*, commonly known as sweet dragon bamboo, is classified within a large cluster of bamboo species in the genus Dendrocalamus belongs to the subfamily, Bambusoideae. It possesses four distinctive characteristics: freshness, sweetness, tenderness, and crispness. It can be enjoyed directly as fresh delicacy, cook it stews after frying, also can be processed into fresh sprout and flavored sprout products (Hui et al., 2019). *D. brandisii* is widely cultivated in the subtropical regions of Southeastern China, particularly in Yunnan Province. Its primarily distribution covers the western and southern areas of Yunnan such as Baoshan, Dehong Dai, and Jingpo Autonomous Prefecture, Lincang, Pu′er City, Xishuangbanna, Red River, and Yuxi (Hui et al., 2022). The yield and quality of *D. brandisii* bamboo shoots are influenced by a variety of factors such as soil, climate, altitude, and fertilization measures.

A population of microorganisms within soil are typically referred to as microbial communities, and these play a vital role in the assimilation of nitrogen and phosphorus by plants (Dini-Andreote et al., 2016). Furthermore, mycorrhizal within these communities further transform soil phosphorus, thereby increasing its absorption ability by plants (Zhu et al., 2018). Soil microbes can affect soil fertility and plant growth in various ways, including soil nutrient status, biomass, and health, which in turn affect the structure and function of soil microbial communities (Chen et al., 2021; Liu et al., 2020). Therefore, in addition to soil chemical properties, plant species characteristics and vitality have a considerable impact on the soil microbial community within the rhizosphere (Xu et al., 2022). The influence of soil pH dissolved organic carbon (DOC), available P (AP), available K (AK), fungi, and bacteria have demonstrated a positive effect on cucumber yield and quality, whereas soil electrical conductivity (EC) has a negative effect (Li et al., 2024). These properties also interact with each other, and there is a strong correlation that indirectly affects cucumber yield and quality (Wang et al., 2021). The study found that the nutritional quality of jujube fruits is influenced by soil nutrients, including organic matter, alkali-hydrolyzed nitrogen, available phosphorus, and pH, and that suitable soil nutrient levels can positively promote the nutritional quality of jujube fruits (Kadir et al., 2017). Soil microorganisms play a central role in the nutrient conversion of crops, and rich microbial diversity in the soil can enhance the accumulation and cycling of nutrients in crops (Pii et al., 2015). Functional microorganisms in the soil, such as those that solubilize phosphorus and fix nitrogen, utilize soil nutrients and atmospheric nitrogen, promote phosphorus solubilization, and facilitate plant hormone synthesis, thereby directly promoting plant growth through various mechanisms (Todeschini et al., 2018). In other crops, it has been found that increasing microbial richness can enhance the nutritional quality of black goji berries; in navel oranges, the total sugars and reducing sugars in the fruit showed a significant positive correlation with Gram-negative bacteria and aerobic bacteria, while vitamin C was significantly negatively correlated with cellulose bacteria (Fibrinobacter) and *Hydrogenobacter thermophilus* (Wang et al., 2024). Research has shown that soil pH, dissolved organic carbon (DOC), available phosphorus (AP), available potassium (AK), and the numbers of fungi and bacteria have positive effects on yield and quality, while soil electrical conductivity (EC) has a negative effect. These properties also interact with each other and exhibit strong correlations, indirectly influencing the yield and quality of cucumbers (Chai et al., 2021). Climatic conditions, such as temperature and precipitation, can affect fruit quality by influencing the soil environment and shaping the community and metabolic activity of soil microorganisms (Chai et al., 2021; Pasternak et al., 2013; Jin et al., 2017). Similar findings have been observed in bamboo, as bamboo quality was determined to be dependent on the composition of the surrounding soil (Shi, 2019). Furthermore, soil texture, type, and fertility are key factors in determining bamboo shoot quality under similar climatic conditions, while changes in soil pH, total nitrogen (TN), and organic matter content of green bamboo (*Dendrocalamopsis oldhami*) from different origins influence reducing sugar, moisture, and total water-soluble sugar in fresh shoots (Zheng, 2012). A positive correlation between starch content of bamboo shoots and soil quick nitrogen content was found in a study of bamboo shoot quality of *Dendrocalamus latiflorus* shootin different landraces (Yu et al., 2020). Furthermore, bamboo shoot ash was significantly positively correlated with the total available phosphorus and hydrolyzed nitrogen and significantly negatively correlated with TN and magnesium (Yang et al., 2021).

Different geographical provenances are affected by diverse environmental factors, the growth conditions, yield and quality of *D. brandisii* shoots may vary significantly across different regions. Therefore, it is important to study how the soil fertility to maintain the yield and quality of *D. brandisii* shoots. However, the characteristics of soil microorganisms in different geographical environments remain unclear, and the relationship between soil microbes, environmental factors, and *D. brandisii* bamboo shoot quality has not been confirmed. This study focuses on the soils of sweet bamboo from different geographic sources in Yunnan Province, examining the soil nutrients and microbial communities associated with these sources. The objectives are (1) explore the relationships between soil microbial communities and their composition in sweet bamboo forests from different geographic sources. (2) Determine the relationships among soil microorganisms, soil properties, and the nutritional quality of bamboo shoots as influenced by different geographic sources. The aim is to provide a scientific theoretical reference for the efficient cultivation of sweet bamboo, assessment of soil conditions, and regulation of microenvironments.

## 2 Materials and methods

### 2.1 Study area profile

The sampling locations in the study area were primarily chosen from the main distribution regions of sweet bamboo: the sampling site in Baoshan Changning (CN) is characterized by a subtropical monsoon climate, with an average annual temperature of 14.9°C, average annual precipitation of 1,259 mm, and a frost-free period of 253 days; the sampling site in Cangyuan (CY), located in southeastern Lincang, has a subtropical low-latitude mountain monsoon climate, with an average annual temperature of 17.2°C, average annual precipitation of 1,595 mm, and 2,115 h of sunshine per year; the sampling site in Mangshi Town, Dehong (MS) has a South Asian subtropical monsoon climate, with an average annual temperature of 19.6°C and average annual precipitation of 1,476 mm; the sampling site in Mingtai Township, Lincang (MT) has a subtropical mountain monsoon climate, with an average annual temperature of 12.2°C and average annual precipitation of 1,300 mm; the sampling site in Simao District, Pu′er (SM) belongs to a low-latitude plateau South Asian subtropical monsoon climate, characterized by high latitude, high temperatures, abundant rainfall, humidity, and calm winds, with an average annual temperature of 18.9°C and average annual precipitation of 1487.5 mm; the site in Shiping County, Honghe (SP) has a subtropical plateau mountain monsoon climate, with an average annual temperature of 18.3°C, average annual precipitation of 961.5 mm, and an average annual relative humidity of 75%; the sampling site in Xinping County, Yuxi (XP) has a subtropical monsoon climate with ample sunlight throughout the year, an average annual temperature of 18.9°C, and average annual precipitation of 1487.5 mm. The basic conditions of the seven sweet bamboo planting sites are summarized in Table 1. All seven sampling sites consist of artificially cultivated pure forest communities, managed extensively, with the collected subjects being sweet bamboo shoots and the underlying soil; sampling took place from August 16 to September 8, 2022.

**Table 1:** Basic situation table of sampling sites.

Sampling location	Longitude	Latitude	Altitude	Slope	Soil type	Slope direction
CN	99°39′48″	24°31′32″	1,229 m	10°	Yellow soil	Western Slope
CY	99°14′43″	23°11′47″	1,425 m	13°	Yellow-brown soil	Southwest Slope
MS	98°23′17″	24°26′37″	1,022 m	13°	Yellow-brown soil	South Slope
MT	100°16′35″	23°45′57″	1,051 m	15°	Yellow soil	western Slope
SM	101°8′5″	22°44′17″	1,236 m	17°	Lateritic red soil	southwest Slope
SP	102°26′49″	23°44′22″	1,457 m	10°	Lateritic red soil	western Slope
XP	101°35′21″	24°0′28″	726 m	11°	Yellow-brown soil	Southwest Slope

Modified from Chen et al. (2024), Agronomy 2024, 14, 2010. https://doi.org/10.3390/agronomy14092010. Licensed under CC BY 4.0.

### 2.2 Test method

#### 2.2.1 Collection of soil samples

In each sampling area, five clumps of *D. brandisii* were selected as samples using the “Five-point method.” The soil surface mulch was removed, and soil samples were collected from the 0–20 cm soil layer. These soil samples were thoroughly mixed to ensure homogeneity. The mixed soil sample was then divided into two parts. One part was packed into a 5 mL sterile centrifuge tube and promptly transferred to the laboratory (−80°C) under dry ice preservation. Upon arrival, it was stored in an ultra-low temperature refrigerator at −80°C. The remaining portion of the samples was stored in sterile sealed bags and transported back to the laboratory in a 4°C refrigerator for subsequent chemical property testing. All sampling procedures were conducted under aseptic conditions.

#### 2.2.2 Bamboo shoot sample collection and processing

Bamboo shoots sampling selection of soil sampling point next to the bamboo clumps out of the soil 30 cm, size based on uniformity, no pests and diseases affect the shoots, shoots without obvious hollow, no deformity, not dry shrinkage of *D. brandisii* bamboo shoots, bamboo shoots dug out intact, each sample of the ground to take 3 bamboo shoots, a total of 21 shoots samples.

The weight (g), length (cm) and base diameter (cm) of each shoot were measured immediately after sampling. Remove the shell of the bamboo shoot and weigh it, calculate the edible rate (%) of the shoot; The bamboo shoots were sliced lengthwise, placed on the prepared tin foil, and put into the oven. The bamboo shoots were killed at 120°C for 30 min, then turned to 75°C at constant temperature and baked to constant weight for the determination of nutritional indexes.

#### 2.2.3 Determination of soil chemical properties

Soil pH was measured using a pH meter (FE20K, Mettler-Toledo, Switzerland) at a ratio of 2.5:1 (water: soil) (Zhang et al., 2023). Soil organic matter (SOM) was determined by the hydrothermal potassium dichromate oxidation colori-metric method (Zhang et al., 2023). Soil total nitrogen (TN) was determined using the Kjeldahl distillation method (Jones and Willett, 2006). Soil total phosphorus (TP) was determined by NaOH fusion, Mo–Sb colorimetry, and ultraviolet spectrophotometry (UV2600, Shimadzu, Japan) (Miranda et al., 2001). Soil available (AP) was analyzed by sodium bicarbonate extraction molybdenum antimony resistance colorimetry (Zhang et al., 2023). Soil total potassium (TK) was determined with a flamephotometer (FP6410, China) (Zhang et al., 2023). Available potassium (AK) was measured by NH4CH3CO2extraction and atomic absorption spectrometry (Zhang et al., 2023).

#### 2.2.4 Soil DNA extraction, sequencing, and amplification

Total genomic DNA was extracted from DNA samples using the MagAttract PowerSoil Pro DNA Kit (Qiagen), with subsequent assessment of DNA quality via agarose gel electrophoresis, and determination of DNA concentration and purity using NanoDrop2000. For the amplification of the V3-V4 variable region of the 16S rRNA gene, upstream Primer 338F (5′-ACTCCTACGGGGAGGCAGCAG-3′) and downstream primer 806R (5′-GGACTACHVGGGGTWTCTAAT-3′) (Liu et al., 2016) were utilized. The ITS1F region (ITS1F, CTTGGTCATTTAGGAAGTAA; ITS2R, GCTGCGTTCTTCATCGATGC) of the rRNA gene was amplified using ITS1F/ITS2R primers. The PCR reaction system (20 μL) comprised 10 × Buffer (2 μL), 2.5 mM dNTPs (2 μL), Forward Primer (5 μM) (0.8 μL), Reverse Primer (5 μM) (0.8 μL), rTaq Polymerase (0.2 μL), BSA (0.2 μL), Template DNA (10 ng), with ddH2O added to make up the total volume to 20 μL. Amplification involved an initial predenaturation step at 95°C for 3 min, followed by 30 cycles (95°C denaturation for 30 s, 55°C annealing for 30 s, 72°C extension for 45 s), with a final extension at 72°C for 10 min, and storage of the products at 4°C. Each sample underwent 3 replicates. A library of purified PCR products was constructed using the NEXTFLEX Rapid DNA-Seq Kit, with 3 replicates per sample. PCR products from the same samples were pooled and recovered on a 2% agarose gel, followed by purification using the AxyPrep DNA Gel Extraction Kit (Axygen Biosciences, Union City, CA, USA). The purified products were then subjected to electrophoresis on a 2% agarose gel and quantified using the Quantus™ Fluorometer (Promega, USA). Sequencing was performed using Illumina’s Miseq PE300/NovaSeq PE250 platform (Shanghai Meiji Biomedical Technology Co., Ltd.), and the raw data were uploaded to the NCBI SRA database (serial number: PRJNA1060609).

### 2.3 Statistics and analysis of data

Raw sequencing data underwent quality control and sequence merging using Fastp (0.19.6) (Chen et al., 2018) and Flash (1.2.11) (Magoč and Salzberg, 2011). Subsequently, sequences were filtered and chimeras removed utilizing UPARSE (7.1) (Edgar, 2013; Stackebrandt and Goebel, 1994), with OTU clustering performed at 97% similarity. Annotation was conducted using RDP (2.11) (Wang et al., 2007) against the Silva 16S rRNA gene database (V138) and Unit database (V7.2), employing a confidence threshold of 70%. Mothur software (Schloss et al., 2009)^1^ was employed to calculate Chao 1, Shannon, and other biodiversity indices, with between-group differences in Alpha diversity analyzed using the Wilxocon rank-sum test. Principal coordinate analysis (PCoA) based on the Bray-Curtis distance algorithm was utilized to assess the structural similarity of microbial communities among samples. Additionally, the Permanova nonparametric test was employed to analyze significant differences in microbial community structure among samples. The LEfSe analysis (Linear discriminant analysis Effect Size) (Segata et al., 2011)^2^ was utilized to identify significant differences between samples, with the LDA set at 4. Distance-based redundancy analysis (db-RDA) was conducted to investigate the relationship between microbial communities and soil chemical properties, as well as the nutritional quality of bamboo shoots. The db-RDA analysis, based on the Bray distance algorithm, aimed to explore the correlation between microbial communities and soil chemical properties and bamboo shoot nutritional quality. Data were organized and calculated using Excel 2010, and statistical analysis was performed using SPSS (26.0). One-way ANOVA was used to assess the significance of differences in soil chemical properties and the nutritional quality of bamboo shoots, while Duncan’s analysis method was employed for fuzzy membership function evaluation.

## 3 Results

### 3.1 Chemical properties of the soil

There is a wide variation in soil chemical properties among the different geographic seed sources of *D. brandisii*, as shown in Table 2. The seven main chemical indices differed significantly (*p* < 0.05) between the different regions. The pH value of soil in SP was significantly higher than that in CY (*p* < 0.05), and significantly higher than that in the CN, MS, MT, and SM (*p* < 0.01), SM soil was observed to be the most acidic. In addition to CN, MS and SM, the other four areas had higher soil nutrients, and the organic matter content in CY was significantly higher than that in MS, MT, and SM (*p* < 0.01). The content of soil AP in MT was the highest, and the difference was very significant (*p* < 0.01); the content of soil AP in the SP area was significantly higher than that in CN, MS, CY, and SM (*p* < 0.01). The TN content in XP was significantly higher than that in MS, MT, and SM (*p* < 0.05), and the lowest was observed in the MS. The content of total phosphorus in MT was the highest, followed by that in CY (*p* < 0.01), and was significantly higher than that in CN, MS, SM, SP and XP. The soil total potassium content in XP was the highest, and the difference was significantly higher than that in the other six regions.

**Table 2:** Soil chemical properties in different *D. brandisii* geographical provenances.

Soil factor	CN	CY	MS	MT	SM	SP	XP
pH**	5.13 ± 0.13bc	5.56 ± 0.20bc	4.92 ± 0.15bc	4.74 ± 0.01 cd	4.33 ± 0.09d	6.50 ± 0.67a	5.80 ± 0.05ab
SOM*(g·kg^−1^)	49.24 ± 4.47ab	64.83 ± 8.99a	32.08 ± 3.89bc	30.78 ± 1.49bc	23.64 ± 2.44c	43.19 ± 2.55abc	53.10 ± 16.26ab
AP**(mg·kg^−1^)	14.43 ± 6.01b	5.88 ± 0.65b	3.24 ± 0.33b	57.85 ± 12.07a	13.80 ± 2.82b	3.67 ± 0.40b	5.71 ± 3.55b
AK**(mg·kg^−1^)	59.58 ± 15.21c	205.83 ± 54.95bc	68.33 ± 9.82c	361.67 ± 127.33ab	172.50 ± 62.37bc	498.33 ± 60.85a	372.08 ± 74.74ab
TN**(g·kg-^1^)	2.13 ± 0.55ab	3.41 ± 0.63a	1.76 ± 0.27c	1.79 ± 0.10c	1.78 ± 0.29c	2.75 ± 0.15ab	3.45 ± 0.74a
TP**(g·kg^−1^)	0.56 ± 0.03bc	0.90 ± 0.05a	0.41 ± 0.02bc	0.97 ± 0.12a	0.38 ± 003c	0.46 ± 0.01bc	0.62 ± 0.12b
TK**(g·kg^−1^)	10.09 ± 0.74d	17.59 ± 2.81bc	19.09 ± 1.15b	14.20 ± 0.54 cd	21.99 ± 0.98b	5.67 ± 0.31e	28.96 ± 1.58a

Values are mean ± SE; different letters in the same column indicate significant differences (*p* < 0.05), * and ** indicate significant differences at the 0.05 and 0.01 level, respectively. CN: Changning County, Baoshan City; CY: Cangyuan County, Lincang City; MS: Mang County, Dehong Prefecture; MT: Matai Township, Linxiang District, Lincang City; SM: Simao District, Pu’er City; SP: Shiping County, Honghe Prefecture; and XP: Xinping County, Yuxi City. SOM: soil organic matter; AP: available phosphorus; AK: available potassium; TN: total nitrogen; TP: total phosphorus; TK: total potassium. Modified from Chen et al. (2024), Agronomy 2024, 14, 2010. https://doi.org/10.3390/agronomy14092010. Licensed under CC BY 4.0.

### 3.2 Nutritional quality of bamboo shoots

**Figure 1:**
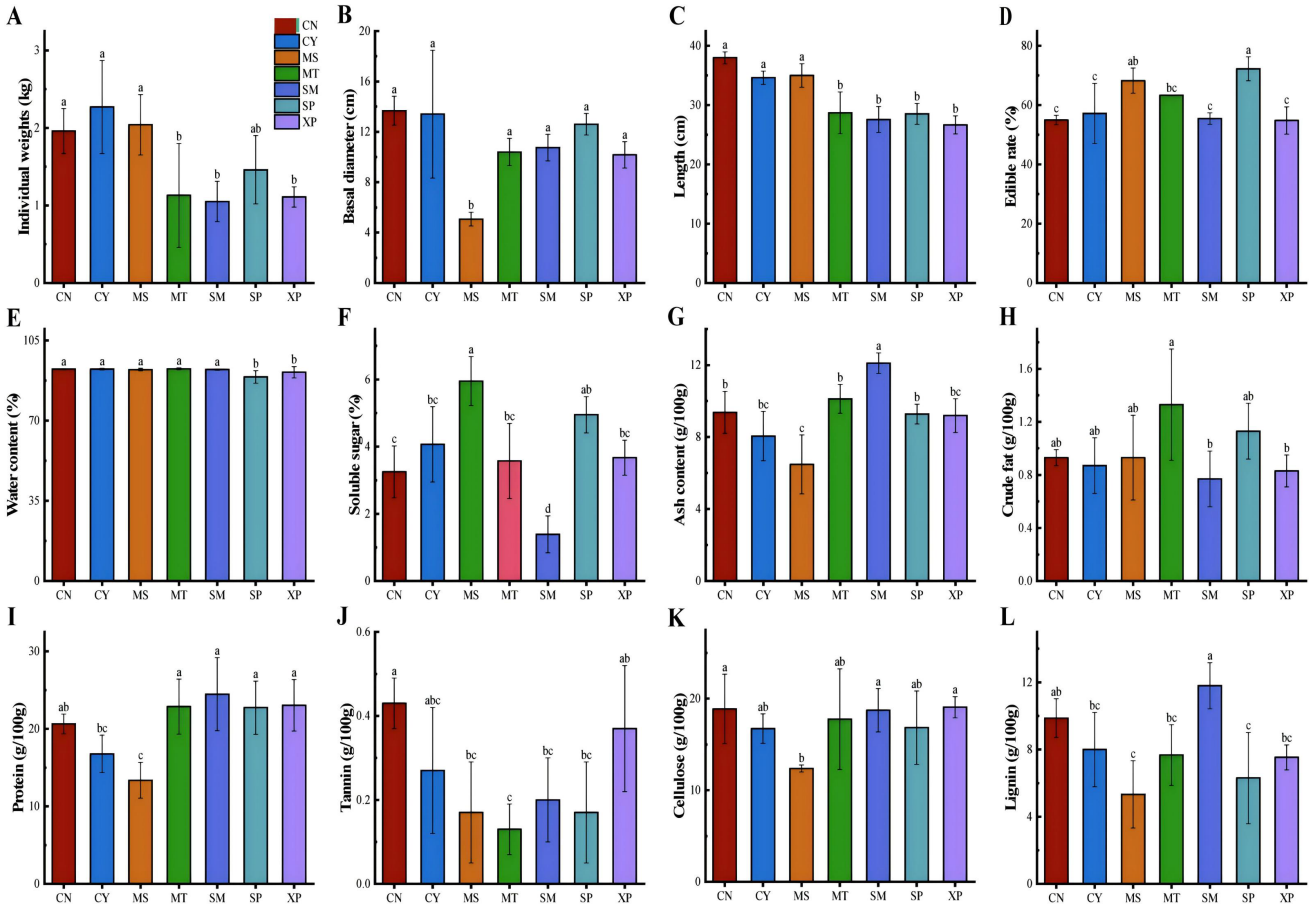
Analysis of *D. brandisii* bamboo shoot quality from different geographical provenances based on **(A)** individual weight, **(B)** basal diameter, **(C)** length; **(D)** edible rate, **(E)** water content, **(F)** soluble sugar, **(G)** ash content, **(H)** crude fat, **(I)** protein, **(J)** tannin content, **(K)** cellulose, and **(L)** lignin. The different letters indicated that there were significant differences in shoot quality among different regions (*p* < 0.05).

Four appearance (individual weight, basal diameter, length, and edible rate) and eight nutritional quality indices (water content, soluble sugar, ash, crude fat, protein, lignin, cellulose, and tannin) were selected to measure the quality of *D. brandisii* bamboo shoots, and the results are shown in Figures 1A–L. The appearance quality showed that the individual weight of the bamboo shoots was highest in CY, followed by MS and CN, which were significantly (*p* < 0.05) higher than those of bamboo shoots from MT, SM, and XP. The basal diameter of bamboo shoots in MS was highly significant (*p* < 0.01) in CN, CY and SP; the length of bamboo shoots in CN was the largest and highly significant (*p* < 0.01) and was higher than that of MT, SM, SP, and XP, and was not significantly different from CY and MS. The edible rate of bamboo shoots was highly significant (*p* < 0.01) in SP. There were significant differences in the appearance and morphology of *D. brandisii* bamboo shoots grown in natural environments of different geographies, and there were no significant correlations between shoot length, weight, basal diameter, and palatability.

**Figure 2:**
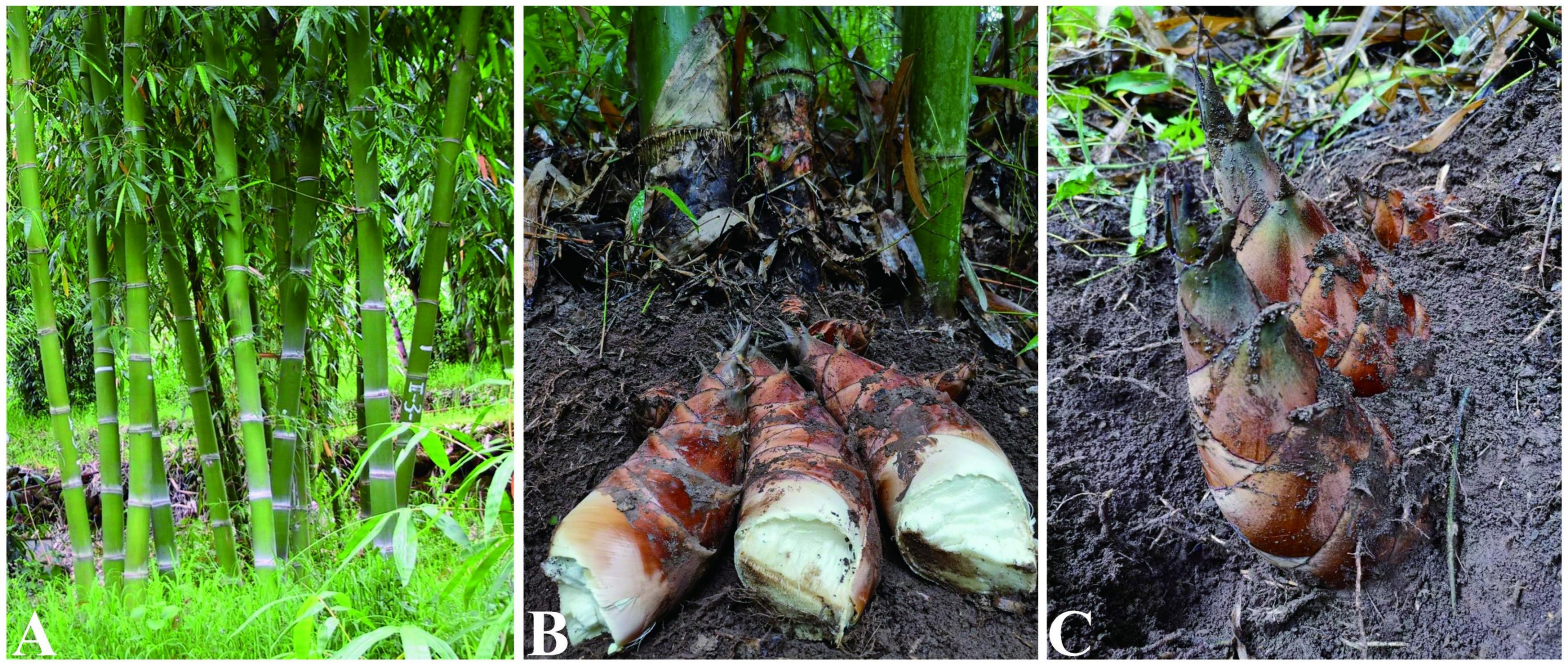
Morphological characterization of *D. brandisii*. Morphology of *D. brandisii* forest **(A)**. Morphology of *D. brandisii* bamboo shoots **(B,C)**. Photography by Hui chaomao.

Nutritional quality of *D. brandisii* bamboo shoots in SP and XP had significantly (*p* < 0.05) lower water content than CN, MS, CY and MT. The soluble sugar content was significantly higher in MS (*p* < 0.01) and CY (*p* < 0.05) than compared to CN, MT, SM, and XP. The ash content of MS (*p* < 0.01) bamboo shoots was significantly lower than that of CN, MT, SM, SP, and XP, whereas CY (*p* < 0.05) was significantly lower than that of MT and SM. The crude fat content of MT bamboo shoots was significantly (*p* < 0.05) higher than that of CY, SM and XP; SM bamboo shoots had the highest protein content, which was not significantly different from that of MT, SP, and XP, and MS had significantly (*p* < 0.05) lower content than CN, MT, SM, SP, and XP, lignin was highly significant (*p* < 0.01) in SM bamboo shoots than in MS and SP, and significantly higher (*p* < 0.05) than in CY, MT, and XP; XP bamboo shoots had the highest cellulose content, which was significantly different from CN, SM and XP. The tannin content of CN bamboo shoots was significantly (*p* < 0.05) higher than that of MS, MT, SM, and SP. Overall, the nutrient composition of *D. brandisii* bamboo shoots from different geographic seed sources varied considerably, with different degrees of variation in different nutrient components, with the nutritional quality of *D. brandisii* bamboo shoots in CN being better on the whole. The CN regional seed source has been approved by the Forestry and Grassland Bureau of the State Forestry and Grassland Bureau of the Forestry and Tree Species Validation Committee on January 25, 2022, “Yun Sweet No. 1” Sweet Longzhu as a national grade good seed, good seed number: State S-SV-DB-016-2021 (with Figure 2).

Based on the measurement results of various indicators for sweet bamboo shoots, the outcomes of the fuzzy membership analysis are shown in Table 3. The average values of the membership function across the seven regions are ranked as follows: CN > CY > MT > XP > SM > SP > MS. This indicates that the nutritional quality of sweet bamboo shoots from the CN region is overall the best, while the nutritional quality in the Shiping and Mangshi regions is relatively poor.

**Table 3:** Membership degree of nutrient content in bamboo shoots.

Indicator	CN	MS	CY	MT	SM	XP	SP
Moisture content	0.9768	0.9429	0.9779	1.0000	0.9474	0.4024	0.0000
Soluble sugars	0.4072	1.0000	0.5870	0.4773	0.0000	0.7800	0.4986
Ash content	0.5137	0.0000	0.2798	0.6465	1.0000	0.4983	0.4811
Crude fat	0.2940	0.2940	0.1765	1.0000	0.0000	0.6470	0.1175
Protein	0.6431	0.0000	0.3009	0.8407	1.0000	0.8289	0.8555
Lignin	0.7028	0.0000	0.4124	0.3609	1.0000	0.1495	0.3402
Cellulose	0.9701	0.0000	0.6517	0.8060	0.9502	0.6667	1.0000
Tannin	1.0000	0.1113	0.4447	0.0000	0.2223	0.1113	0.7780
Individual weight	0.7466	0.8147	1.0000	0.0708	0.0000	0.3352	0.0518
Base diameter	1.0000	0.0000	0.9690	0.6202	0.6608	0.8760	0.5930
Length	1.0000	0.7587	0.7292	0.2520	0.0000	0.2386	0.0885
Edibility rate	0.0083	0.7698	0.1360	0.4863	0.0367	1.0000	0.0000
Mean value	0.6885	0.3910	0.5554	0.5467	0.4848	0.5445	0.4003
Ranking	1	7	2	3	5	4	6

### 3.3 Diversity of soil microbial community

A total of 910,389 bacterial sequences were obtained from 21 soil samples collected from seven provenances, which were effectively classified into 6,885 operational taxonomic units (OTUs) with 97% similarity, comprising of 2096 species belonging to 1,001 genera, 511 families, 308 orders, 127 classes, and 40 phyla. The total number of available sequences of soil fungi was 903,506, which can be divided into 7,021 OTUs, including 1,627 species, 901 genera, 375 families, 156 orders, 16 phyla, and 63 classes. A one-way analysis of variance (ANOVA) was performed for bacterial and fungal communities in the soil samples.

**Figure 3:**
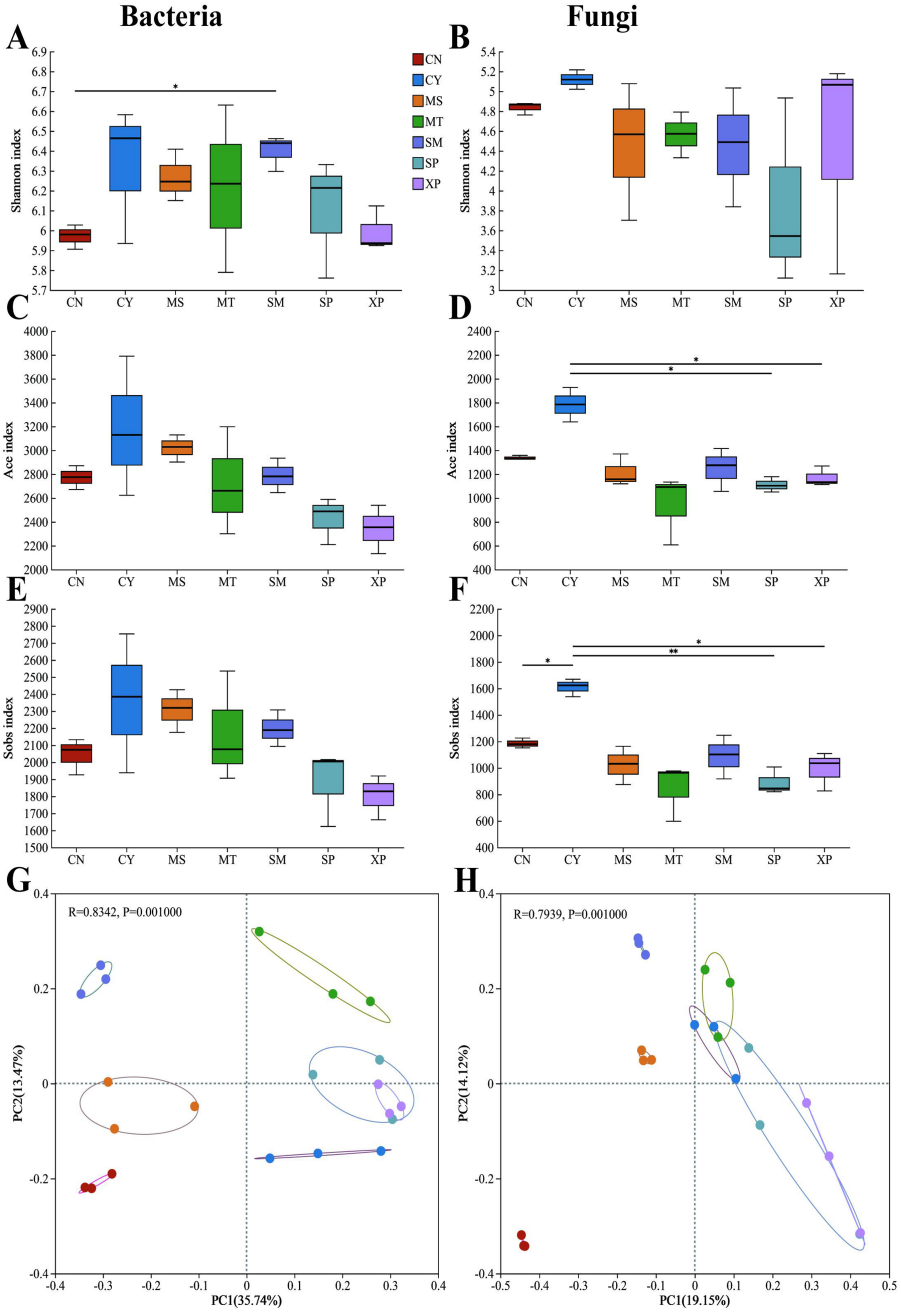
*α* and *β* diversity of microbial communities in different soil samples. Shannon indices, ACE indices, Sobs indices, and PCoA of bacterial **(A,C,E,G)** and fungal **(B,D,F,H)** communities, respectively. PCoA: The principal coordinate analysis (PCoA) based on Bray-Curtis distance; **(A–F)** * and ** indicate significant differences at the 0.05 and 0.01 level, respectively.

The soil microbial community diversity index of different geographical provenances showed a significant difference (*p* < 0.05) when comparing the *α* diversity of a microbial community (Figures 3A–F), in the bacterial community, the Shannon index was significantly different between SM and CN (*p* < 0.05), the ACE index of fungi community was significantly different from that of SP and XP (*p* < 0.05), CY in Sobs index was significantly different from CN and XP (*p* < 0.05) and highly significantly different from SP (*p* < 0.01). Principal coordinate analysis (PCoA) of the bacterial and fungal communities showed that the microbial communities in the soil samples from different geographical *D. brandisii* provenances were significantly different. Specifically, fungal PCoA (Figure 3H) exhibited more pronounced clustering between samples compared with bacterial PCoA (Figure 3G), and the soil fungal communities in the CN region were more distant and different from those in the other 6 regions. Both SP and XP bacteria and fungi showed clusters, and the species composition of the microbes in the two samples was similar. In addition, analysis of similarities (ANOSIM) showed that different geographic provenances had significant effects on the structure of soil bacteria (*r*^2^ = 0.8342, *p* = 0.001) and fungal communities (*r*^2^ = 0.7939, *p* = 0.001) (Figures 3G,H).

### 3.4 Soil microbial composition

**Figure 4:**
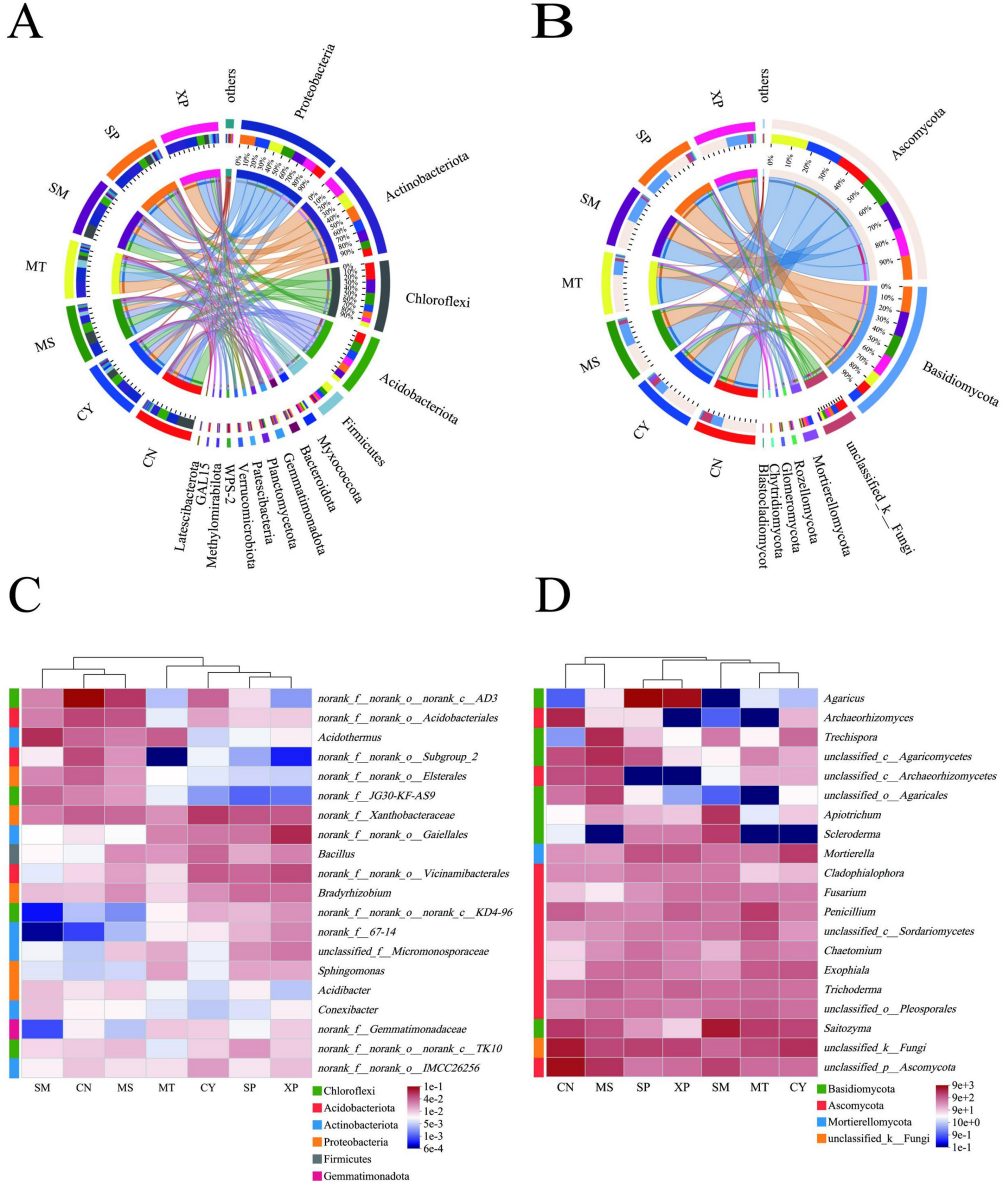
Microbial community composition in soil samples, abundance of phyla major bacterial **(A)** and fungal **(B)** communities. The heat map shows the top 20 abundant genera of bacteria **(C)** and fungi **(D)**.

The soil bacteria in the seven regions belonged to 998 genera, 509 families, 307 orders, 127 classes, and 40 phyla, as shown in Figures 4A–D. Proteobacteria (19.78–29.06%), Actinobacteria (13.53–30.01%), Chloroflexi (8.03–31.47%), and Acidobacteria (7.12–19.17%) were the dominant Chloroflexi in the soil bacterial community and together accounted for more than 70% of the total bacterial community in each soil sample (Figure 4A and Supplementary Table S1). The relative abundance of Proteobacteria was the highest in SP (29.06%) and lowest in CN (19.78%); the relative abundance of Actinobacteria was the highest in XP (37%) and lowest in CN (13.53%); and the relative abundance of Campylobacter was the highest in CN (31%) and lowest in MT (8.03%). The dominant bacterial genera in all soil samples were *Xanthobacteraceae* (2.83–7.29%), *AD3* (0.35–13.68%), *Acidothermus* (0.62–8.06%), and *Gaiellales* (0.94–8.64%), respectively (Figure 4C and Supplementary Table S2).

Soil fungi in the seven regions belong to 901 genera, 375 families, 156 orders, 63 classes, and 16 phyla, and the dominant fungal phyla were Ascomycota (41.72–62.44%) and Basidiomycota (18.72–45.23%), which accounted for more than 79% of the total fungal community in each soil sample (Figure 4B and Supplementary Table S3). The MT (65.01%) and CY (62.44%) regions had the highest relative abundances of ascomycetes, whereas SP had the lowest (41.72%). However, SP had the highest relative abundance of Ascomycota (45.23%), and CY had the lowest (18.72%). The dominant fungal genera of the seven geographic soil samples varied, with *unclassified_p_Ascomycota* and *Archaeorhizomyces* in CN, *Mortierella* and *Exophiala* in CY, *unclassified_c_Agaricomycetes* and *Trechispora* in MS, *Penicillium* and *unclassified_c_Sordariomycetes* with the highest abundance in MT, *Saitozyma* and *Apiotrichum* in SM, and *Agaricus* in SP and XP (Figure 4D and Supplementary Table S4). This result suggests that the microbial community structure varies significantly under natural conditions in different geographies and that fungal communities are more influenced by the environment.

### 3.5 Analysis of soil microbial abundance

**Figure 5:**
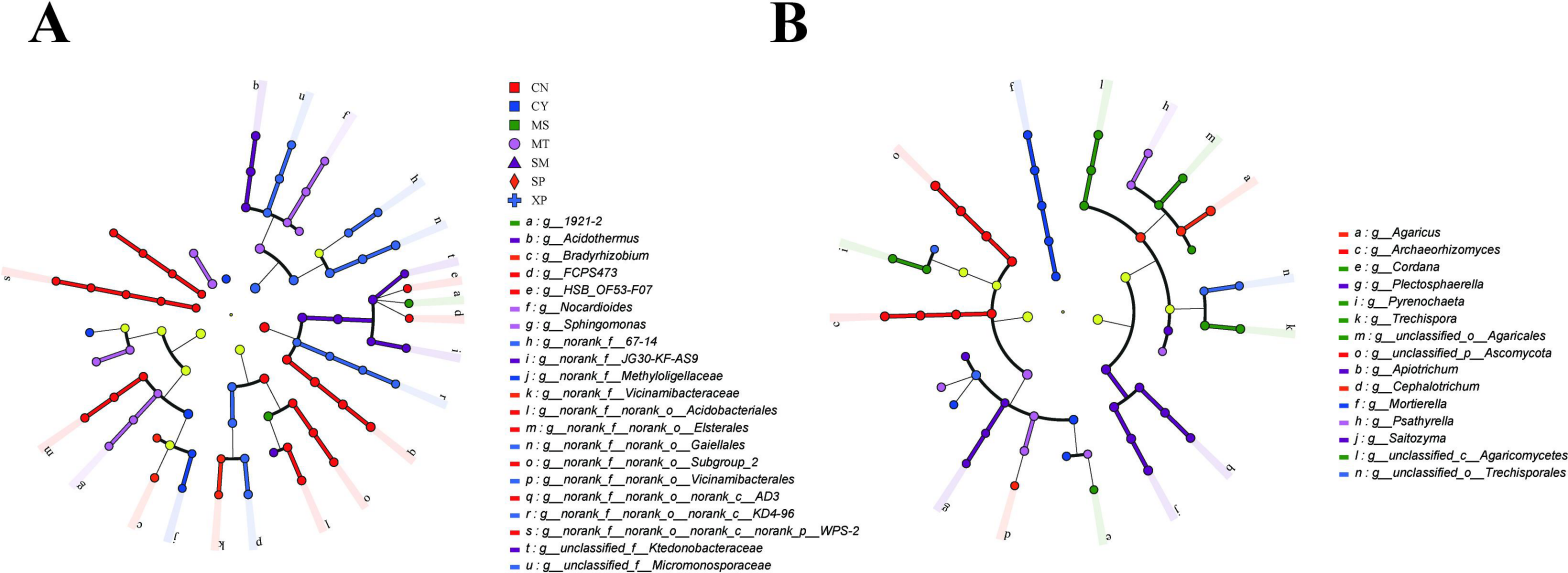
LEfSe analysis of microbial abundance in soil samples from the seven districts showing taxa with different abundance values for bacterial **(A)** and fungal **(B)** communities. LEfSe: linear discriminant analysis effect size.

Using the linear discriminant analysis effect size (LEfSe), the 21 most abundant microbial taxa with significant differences in abundance between genera were identified in the soil samples (Figures 5A,B). This analysis revealed 21 taxonomic branches exhibiting different abundances of bacterial biomarkers (Figure 5A and Supplementary Table S5). The CY and MS samples had the least number of bacterial taxa, with significant differences in one genus each (*Methyloligellaceae* and *1921–2*, respectively), followed by MT and SP, with significant differences between the two genera. In contrast, soil samples from site CN showed the highest number of bacterial taxa, including *AD3*, *Acidobacteriae*, *Subgroup_2, Elsterales*, *HSB_OF53-F07*, *WPS-2*, and *FCPS473*. Meanwhile, a total of 15 evolutionary clades of fungi with significant differences in abundance were identified in this study (Figure 5B and Supplementary Table S6). Among them, soil samples from MT, XP, and CY had the least number of fungal taxa, with only one genus demonstrating notable variation, namely *Psathyrella*, *Trechisporales*, and *Mortierella*, followed by CN and SP, with two genera. In contrast, soil samples from MS contained the highest number of fungal taxa, including five genera: *Trechispora*, *Agaricomycetes*, *Pyrenochaeta*, *Agaricales*, and *Cordana*.

### 3.6 Relationship between soil chemical properties, microbial communities, and bamboo shoot quality

**Figure 6:**
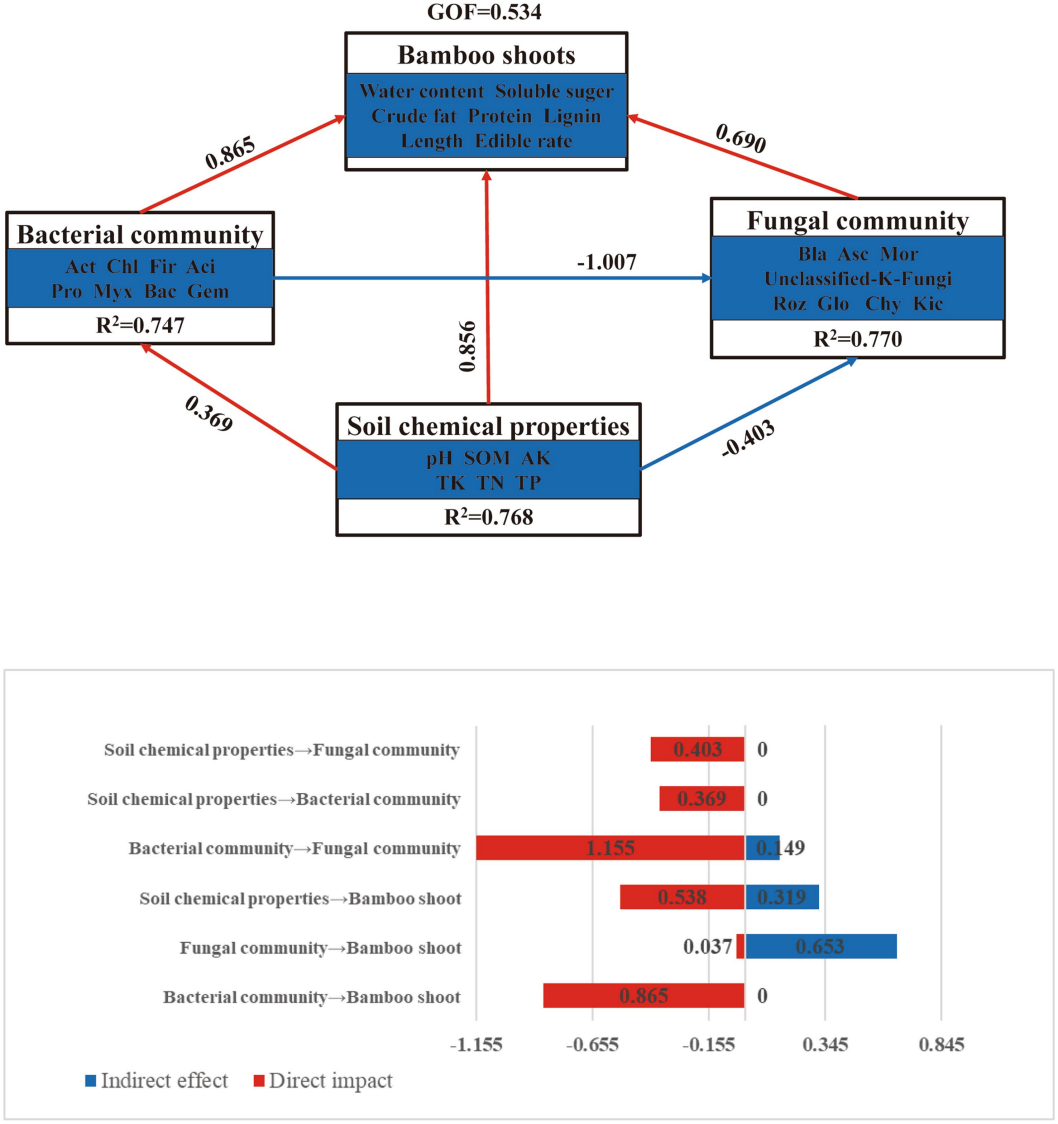
Analysis of partial least squares path modeling analysis of pathways, direct effect coefficients, and standardized total effects of direct and indirect effects of major soil chemical properties and microbial community structure affecting the quality of bamboo shoots of *D. brandisii*. The main soil chemical properties (PH, SOM, AK, TN, TP), which were screened by PLS-PM analysis, revealed that the microbial communities were structured as the first and second axes of bacterial and fungal PCoA. Act: Actinobacteriota; Chl: Chloroflexi; Fir: Firmicutes; Aci: Acidobacteriota; Pro: Proteobac-teria; Myx: Myxococcota; Bac: Bacteroidota; Gem: Gemmatimonadota; Bla: Blastocladi-omycota; Asc: Ascomycota; Mor: Mortierellomycota; Roz: Rozellomycota; Glo: Glomeromy-cota; Chy: Chytridiomycota; Kic: Kickxellomycota. Path coefficients (direct effects) are indicated by arrows, arrows with positive and negative coefficients are indicated in red and blue, respectively, and GOF denotes the model goodness-of-fit index.

The PLS-PM analysis of the soil chemical properties, microbial community structure, and bamboo shoot quality was performed, and the results are shown in Figures 6A,B. Both soil chemical properties and microbial community structure had direct positive effects on bamboo shoot quality. The direct effect coefficient of the soil bacterial community on *D. brandisii* bamboo shoot quality was 0.865. As shown in Figure 6, the total effect of soil bacterial community (0.865) and soil chemical properties (0.856) on bamboo shoot quality, including direct and indirect effects, was significant; the total effect of fungal community structure on bamboo shoot quality was 0.69, which was less than that of bacterial community structure and soil chemical properties, and the indirect effects of bacterial community and soil chemical properties on bamboo shoot quality was less than that of the fungal community structure.

**Figure 7:**
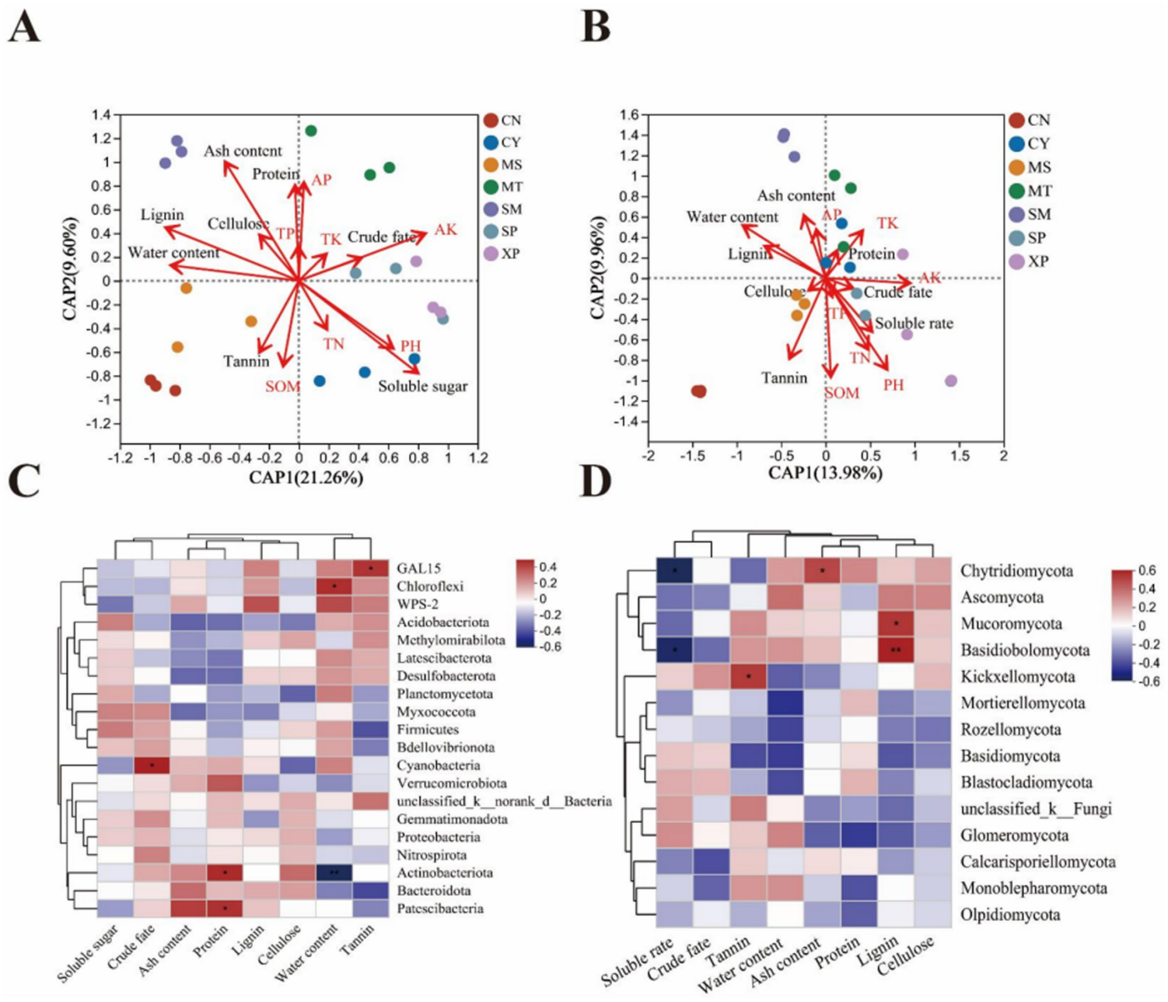
db-RDA analysis plots and correlation thermograms of soil chemical properties, bamboo shoot nutrient quality, and soil microbial abundance in seven regions. Different taxa of bacteria **(A,C)** and fungi **(B,D)** are shown.

To further explore the relationship among the three, db-RDA analysis was performed on soil microorganisms of *D. brandisii* bamboo from different geographical seed sources to investigate the relationship between soil factors, bamboo shoot quality, and microbial communities. Seven nutrient indicators of bamboo shoots were selected as water content, soluble sugar, ash content, crude fat, protein, lignin, cellulose, and tannin, the results showed that pH, AP, and AK were the main soil properties affecting the bacterial community structure (*r*^2^ = 0.35, *p* = 0.019 for pH, *r*^2^ = 0.325, *p* = 0.022 for AP and *r*^2^ = 0.43, *p* = 0.008 for AK) (Figure 7A and Supplementary Table S7). Soil pH, SOM, AK, and TN were the main factors affecting the fungal community structure (*r*^2^ = 0.669, *p* = 0.001 for pH; *r*^2^ = 0.46, *p* = 0.012 for SOM; *r*^2^ = 0.47, *p* = 0.003 for AK; and *r*^2^ = 0.367, *p* = 0.021 for TN) (Figure 7B and Supplementary Table S8). In particular, soil pH and fast-acting potassium levels affect the structure of both bacterial and fungal communities. Bamboo shoot water content, soluble sugar, ash content, protein, and lignin content were strongly influenced by the bacterial community, whereas bamboo shoot water content and tannin were strongly influenced by the fungal community.

The top 20 species in terms of relative abundance at the bacterial and fungal phyla levels were selected for Pearson correlation analysis, as shown in the heatmap (Figures 7C,D). The major effects on the nutrition of *D. brandisii* bamboo shoots were Actinobacteriota, Chloroflexi, Patescibacteria, GAL15, and Cyanobacteria (Figure 7C). At the level of fungal phyla, those that have a greater impact on the nutrition of bamboo shoots include Basidiomycota, Kickxellomycota, Mucoromycota, unclassified-k-Fungi, and Glomeromycota (Figure 7D). Among them, crude fat, cyanobacteria, and Chytridiomycota, demonstrated a significant positive correlation (*p* < 0.05). Protein was significantly and positively correlated with Patescibacteria and Actinobacteriota, (*p* < 0.05). Water content was significantly and positively correlated with Chloroflexi (*p* < 0.05) and significantly and negatively correlated with Actinobacteriota, (*p* < 0.05); tannin was significantly positively correlated (*p* < 0.05) with GAL15 and Kickxellomycota, Mucoromycota, and unclassified-k-Fungi; lignin and Basidiomycota demonstrated a significant positive correlation (*p* < 0.05). Protein and Glomeromycota showed a significant negative correlation (*p* < 0.05). The soluble rate and Glomeromycota showed significant positive correlation (*p* < 0.05).

## 4 Discussion

### 4.1 Relationship between soil nutrients and soil microorganisms

The structure and diversity of soil microbial communities are not only affected by external factors such as climatic conditions, vegetation types, soil types, and human activities, but are also affected by intrinsic factors such as soil properties and nutrient content. These are closely related to soil microbial growth and are highly sensitive to change, with close relationships and interactions existing between factors (Griffiths et al., 2011; Wang et al., 2024). However, it is difficult to determine the consistent effects of land management on soil microbial ecophysiology because intensive land use and its impact on soil properties such as pH, bulk density, and water content often depend on specific sites and environments (Frey et al., 2013; Six et al., 2006). Previous studies have shown that soil chemical properties affect soil microbial communities. For example, AP, total potassium, pH, and AK are the main factors affecting bacterial communities (Zhou et al., 2022). It was observed that both pH and organic matter influenced the bacterial diversity at different taxonomic levels, and they are environmental factors driving bacterial community distribution (Cui et al., 2023). Available nitrogen, soil organic matter, AK, and pH are the main factors influencing soil fungal community composition (Guo et al., 2022). Total potassium and pH significantly affected the soil fungal community structure and abundance (Zhu et al., 2023). Soil pH and nutrients affected the microbial community diversity (Ying et al., 2017). In this study, the seven sample plots showed notable differences in soil bacterial and fungal community structure and diversity under the same *D. brandisii* seed source, planting years, and agricultural management. The pH of each plot sampled in this study ranged from 4.33 to 6.50, and RDA analysis revealed that soil pH had a substantial effect on the bacterial and fungal communities. The results show that soil pH is a key factor affecting microbial community structure, which is consistent with previous studies on biophysical processes of microbial diversity in soil (Tecon and Or, 2017). A similar finding was determined with AK, indicating that soil microbial community structure was closely related to potassium nutrition, it is also demonstrated that AK is an important factor affecting the change of microbial community structure, which is consistent with studies by Tian et al. (2016) and Zhao et al. (2022). The content and proportion of AK in the soil may be the key to maintaining the stability of soil microbial communities. The sample sites were mainly pure forests of *D. brandisii* with a relatively homogeneous vegetation distribution, and soil-limiting resources had a notable effect on the relative abundance of soil bacteria and fungi (Ding et al., 2021; Wei et al., 2020). Therefore, N, P, and K should be reasonably distributed during the cultivation of *D. brandisii*, and attention should be paid to the application of potash fertilizer.

### 4.2 Effects of soil properties on bamboo shoot quality

Shoot quality is an important research topic in bamboo biology, ecology, and silviculture. Soil plays an important role in bamboo shoot quality in terms of their appearance and nutritional quality, therefore, the factors affecting the quality of bamboo shoots are complex and numerous (Bao et al., 2018; Zheng et al., 2019). The results showed that the effects of different soil nutrient factors on the nutritional quality of bamboo shoots were different; there was a negative correlation between SOM and crude fiber content, whereas a positive correlation between soil pH and soluble sugar content of *D. oldhami* shoots were observed (Zheng, 2012). In this study, the contents of the chemical elements and bamboo shoot quality in the soils of different geographic seed sources of *D. brandisii* were substantially different in the seven regions, and correlation analyses demonstrated that soil nutrients had an effect on the formation of bamboo shoot quality, especially soil pH, AK, and TN. Soil AK was significantly negatively correlated with water content, individual weight, and highly significantly negatively correlated with the length of *D. brandisii* bamboo shoots. Soil TN and bamboo shoot water content were significantly negatively correlated. There was a significant negative correlation between TK in soil and the edible rate of bamboo shoot. In this regard, bamboo shoots with special nutritional qualities can be cultivated by changing the content of certain nutrient elements in the soil during the cultivation process of *D. brandisii* in order to improve the quality of bamboo shoots. The content of TN and organic matter in the soil mainly affects the crude fiber content of bamboo shoots, and the high content of AK and total K in the soil is beneficial for the absorption and accumulation of K and P in bamboo shoots (Li et al., 2017; Cao et al., 2021). Therefore, soil factors have an important influence on differences in the nutritional quality of bamboo shoots of *D. brandisii* in different origins. It is inferred that the difference in bamboo shoot quality is coordinated by environmental factors, and different nutrient quality indices are affected by soil nutrient factors; the same nutrient factors affect different nutrient quality indices of bamboo shoots, and there are complex and interactive effects between soil nutrients and bamboo shoot nutritional quality.

### 4.3 Effect of soil microorganism on bamboo shoot quality

Soil bacterial and fungal communities provide nutrients for plant growth and play important roles in soil metabolism, organic matter decomposition, nutrient cycling and transformation, and the balance of soil nutrient supply, further affecting fruit quality. In this study, there were considerable differences observed in the soil microbial structure of *D. brandisii* in the seven districts, including Actinobacteria, Chlorflexi, Firmicutes, Xanthobacteriaceae, and Ktedonobacteraceae, which are the bacterial taxa Mortierellomycota, Glomeromycota, Blastocladiomycota, Agaricaceae, Trimorphomycetaceae, and other fungal groups. The dominant soil microbial taxa in the seven districts were Proteobacteria, Actinobacteria, Chloroflexi, Acidobacteria, Ascomycota, and Basidiomycota. Among them, the highest relative abundance of Proteobacteria was found in SP, where the typical oligotrophic bacteria were Acidobacteria and the eutrophic bacteria were Ascomycetes, adapted to soil environments with high nutrient content (Fierer et al., 2012). Therefore, the relative abundances of Aspergillus and Acidobacterium phyla can be used for soil quality assessments (Shao et al., 2019). The relative abundance of Acidobacteria was highest in CN, suggesting that this taxon may be sensitive to soil carbon accessibility or soil pH. It has been shown that this taxon is mainly driven by soil pH and negatively correlated with soil pH (Lauber et al., 2008). Chioroflexi was the dominant species in *D. brandisii* forests and had the highest relative abundance in CN. It can simply utilize organic matter for Chloroflexi heterotrophic growth in a nutrient-rich environment, promote the use of C, N, S, and other elements in the soil cycle, further improving the nutrient content in the soil, but also through dissolved phosphorus to promote the transformation of phosphorus elements and use efficiency, provide phosphorus nutrients for the soil, promote nutrient absorption in the root system, and maintain its balance with the microenvironment (Xian et al., 2020; Guo et al., 2023). Xinping County, Yuxi City (XP) had the highest relative abundance of actinomycetes, indicating it’s low water tolerance (Pointing et al., 2009; Fierer et al., 2012). In this study, Acidobacteria, Proteobacteria, and Actinobacteria were the dominant soil communities in the seven regions, which was consistent with the wide ecological range of Acidobacteria, Proteobacteria, and Actinobacteria. The distribution of characteristics less affected by the environment and changes in environmental characteristics less affected by all three were characterized as dominant communities in soils from different regions (Teng et al., 2017; Xue et al., 2016). Basidiomycetes degrade lignin, which can be used as an indicator of soil microbial biomass and soil quality (Yao et al., 2023). Basidiomycetes are abundant in environments with a good soil quality. Bacillus and Clostridium, belonging to the phylum Firmicutes, play important roles in carbohydrate metabolism (Zhang et al., 2016). The family Ktedonobacteraceae is a subfamily of Chloroflexi that plays an important role in maintaining the structure and function of rhizosphere soil bacterial communities (Wang et al., 2018). These enriched bacterial and fungal taxa have some capacity for nutrient cycling or the production of secondary metabolites and play important roles in soil nutrient cycling (Sharrar et al., 2020; Gavriilidou et al., 2022). In this study, bamboo shoot quality was directly and indirectly affected by the production of soil bacterial and fungal communities, which may be due to the formation of a stable relationship between microbial communities and a variety of physical and chemical factors over a long period of time, they play a role in the process of soil substance circulation, and the difference of species in different regions leads to the difference of bamboo shoot quality. The plant–soil-microbe benign interaction can be controlled by fertilization and other measures, such as supplying plant nutrients, changing soil properties, promoting soil microbes to play their roles, and regulating soil microbes; however, further research is needed to improve the quality of bamboo shoots.

## 5 Conclusion

The soil properties and shoots quality of *D. brandisii* from various provenances were determined by utilizing PCR and sequencing techniques, and significant differences were observed in soil nutrient properties, soil microbial community diversity, and species composition across different spatial scale. Soil microbes as a bridging role between soil and plants, and converting nutrients from soil into a form that can be absorbed and utilized by plants. Moreover, they can modify the soil by altering community structure and function, thereby preserving the dynamic equilibrium of the soil microenvironment. This study demonstrates that both soil pH and AK have a simultaneous impact on the structure of soil microbial community. Furthermore, the alterations in soil properties and soil microorganisms from various provenances can account for the notable variations in bamboo shoot quality. The soil bacterial community primarily influenced the contents of water, soluble sugar, ash, protein and ligands in bamboo shoots, while the fungal community mainly impacted the water and tannin contents of bamboo shoots. This study not only identified the core microbial species of *D. brandisii* forest land from different geographical provenances, but also emphasized the significant role of soil factors in plant quality. It is necessary to take into account not only the source of seeds, but also the synergistic interaction between soil properties and soil microorganisms to improve the quality of bamboo shoots.

## Data Availability

The datasets presented in this study can be found in online repositories. The names of the repository/repositories and accession number(s) can be found here: NCBI SRA, accession PRJNA1060609.

## Footnotes

^1^ http://www.mothur.org/wiki/calculators

^2^ http://huttenhower.sph.harvard.edu/LEfSe
